# Gamified Virtual Reality Training for Pneumatic Reduction of Intussusception in Children: Formative Evaluation of a Low-Fidelity Prototype

**DOI:** 10.2196/83661

**Published:** 2026-09-08

**Authors:** Ahmad Nizamuddin bin Jipli, Ahmad M S Elaklouk, Anas Shikha, Ibrahim Edris

**Affiliations:** 1 Creative Computing (CC) programme area, School of Computing and Informatics, Universiti Teknologi Brunei, Jalan Tungku Link Gadong, Bandar Seri Begawan, Brunei-Muara District, BE1410, Brunei Darussalam, 673 2461020; 2 RIPAS Hospital, Bandar Seri Begawan, Brunei Darussalam

**Keywords:** gamification, low-fidelity prototype, pneumatic reduction, intussusception, design science research, curriculum analysis, game design

## Abstract

**Background:**

Gamification, defined as the application of game design principles in nongame contexts, has garnered increasing attention for its potential to enhance motivation and engagement across various fields, including medical education and training. However, there is a lack of virtual reality (VR)–based simulations on pneumatic reduction of intussusception (PRI) in children in Brunei, thereby limiting practical training opportunities for medical students and surgical trainees.

**Objective:**

This study aims to obtain experts’ reviews of proposed gamification guidelines by conceptualizing a gamified training prototype for pneumatic reduction of intussusception.

**Methods:**

The design science research methodology (DSRM) was adopted using an iterative development approach. Guidelines for designing gamified training interventions were first identified through a prior systematic literature review. These guidelines were then implemented in the development of a low-fidelity VR prototype as a proof of concept. A qualitative formative evaluation was conducted through a semistructured focus group discussion with 6 healthcare professional participants involved in the pediatric intussusception process.

**Results:**

Participants’ formative evaluation indicated that the embedded guidelines could assist in providing effective VR simulation for PRI. The low-fidelity prototype was clear, consistent, and well-aligned with PRI procedures and processes by the participants. The prototype showed promise as an intervention to ensure and enhance trainee motivation and engagement. The overall layout was seen as appealing. Results are presented and discussed with detailed feedback and recommendations for further improvement.

**Conclusions:**

This study can be viewed as a contribution to the developing literature on the gamification of medical training by exploring the possibility of applying the guidelines to the conceptual design of a gamified training prototype. The findings indicate that the prototype and its design approach are initially accepted by experts. Subsequent research will consist of creating a high-fidelity prototype and testing it with a larger, more varied sample of participants, including surgeons and medical students, to verify its effectiveness and generalizability.

## Introduction

### Background

Gamification, through the incorporation of game elements such as points, badges, and leaderboards into nongame environments, has been demonstrated to enhance user motivation and engagement, improve academic performance, foster interaction and socialization, and promote the development of autonomous learning skills [1,2].

While gamification is widely implemented and anticipated to expand, further research is necessary to explore its applications in educational contexts [3].

Students demonstrated greater engagement in classrooms that incorporated simulation games than in conventional instructional settings. Unlike one-time, faculty-intensive training, simulation can be integrated into regular learning activities. Virtual reality (VR) provides repeatable scenarios that allow learners to practice safely, make mistakes without risk, and engage in deliberate practice, thereby fostering psychological safety, engagement, autonomous learning, and motivation through gamification [4,5]. Similarly, both VR and augmented reality (AR) have been shown to enhance learner engagement and performance, although no significant correlation has been found between the type of technology used and the stages of medical education [6].

In traditional learning environments, a major challenge is students’ lack of motivation to actively engage in learning, often due to distractions such as smartphones. The effective implementation of gamification strategies can transform lengthy and monotonous lessons into engaging and enjoyable learning experiences [7]. However, many educational games lack intrinsic integration of learning content with game mechanics, highlighting the need for further research to enhance both engagement and learning outcomes [8].

The existing literature is limited because no VR simulations are available for pneumatic reduction. This gap indicates a need for simulation-based tools to help users become familiar with procedures. The use of immersive VR and AR technologies in medical training shows promising results, but a thorough assessment is still needed [6].

Recent studies have demonstrated the effectiveness of gamified VR simulations for preparing medical procedures. Yang et al [9] assessed the effectiveness of gamified VR, passive VR, 360° video, and a traditional educational video for preparing adolescents for magnetic resonance imaging (MRI). Gamified VR resulted in less head motion, the highest preparedness scoring, and was the most preferred training format. Furthermore, Yang et al [10] developed a VR-based MRI simulator to introduce patients to MRI and ultimately reduce scan termination rates. Their study found significantly higher user engagement compared with standard preparatory materials. The study’s outcomes demonstrate how interactive and guided VR systems can enhance procedural preparation and usability in medical contexts.

In the first iteration, a systematic review of 48 papers identified game elements and factors influencing students’ motivation and engagement, informing the development of gamification guidelines. Key elements included personalization (eg, avatars and system customization), authoring tools for tailored learning, and badges as indicators of achievement. Challenges, quests, and varied difficulty levels supported goal-oriented tasks and diverse learning abilities, while multimodal feedback (auditory, visual, haptic, and tactile) guided performance. Appealing design, leaderboards, progression systems, and storytelling further enhanced immersion and motivation. Points, scoring, and quizzes reinforced learning through rewards and feedback, while social support promoted collaboration. Technology such as VR and AR increased engagement through immersive interaction. A comprehensive discussion of these guidelines is provided in our previous work [11].

Thus, this paper documents the first iteration by implementing previously identified gamification guidelines into a low-fidelity prototype as a proof of concept. The prototype was reviewed through a focus group discussion with health care professionals involved in the management of pediatric intussusception to obtain feedback on its design and potential as an engaging VR-based medical training intervention for pneumatic reduction of intussusception (PRI). Therefore, this study aimed to present the design, conduct formative evaluations of the low-fidelity, gamified VR prototype by experts and medical practitioners, and explore its perceived suitability, clinical relevance, and feasibility as a potential training tool in Brunei Darussalam.

### PRI

Intussusception is the leading cause of bowel obstruction in infants and young children. It is a condition characterized by the telescoping of a proximal segment of the intestine (intussusceptum) into a distal segment (intussuscipiens), resulting in obstruction [12]. The typical clinical presentation of intussusception in infants and young children includes episodic, colicky abdominal pain, the presence of “red currant jelly” stools, and a palpable abdominal mass on physical examination. This classic symptom triad is observed in fewer than 25% of cases [13].

As the obstruction progresses and bowel ischemia develops, complications such as dehydration, fever, tachycardia, and hypotension may rapidly arise due to bacteremia and bowel necrosis. Hence, an air or contrast enema is the primary treatment if intussusception is suspected, unless it is not indicated.

Therefore, the key exclusions are intestinal perforation (free intraabdominal air), peritonitis, or persistent hypotension. On the other hand, nonoperative reduction offers benefits such as lower morbidity, reduced costs, and shorter hospital stays [12]. Although surgical treatment may sometimes be required, nonsurgical approaches, including pneumatic and hydrostatic reduction, are now the preferred first-line management strategies [14].

Pneumatic reduction is a widely recognized technique for managing intussusception. It has a higher success rate and a lower complication rate than barium enema and hydrostatic reduction methods [15]. In recent years, pediatric hospitals have shifted to using air- or water-soluble isotonic contrast agents to mitigate the risk of barium peritonitis in cases of intestinal perforation [16,17]. Subsequently, many institutions have implemented pneumatic reduction due to its increased efficiency, enhanced safety, reduced procedural complexity, and minimized radiation exposure duration [18]. The procedure is performed under fluoroscopic guidance, with air introduced into the rectum. Alexandra C. Maki & Mary E. Fallat [12] discussed that to ensure safety, the recommended maximum air pressure is 80 mmHg for younger infants and 110‐120 mm Hg for older infants; moreover, potential limitations of pneumatic reduction include the risk of tension pneumoperitoneum and inadequate visualization of lead points or the intussusception reduction process, which may lead to false-positive outcomes.

## Methods

### Overview

**Figure 1.**
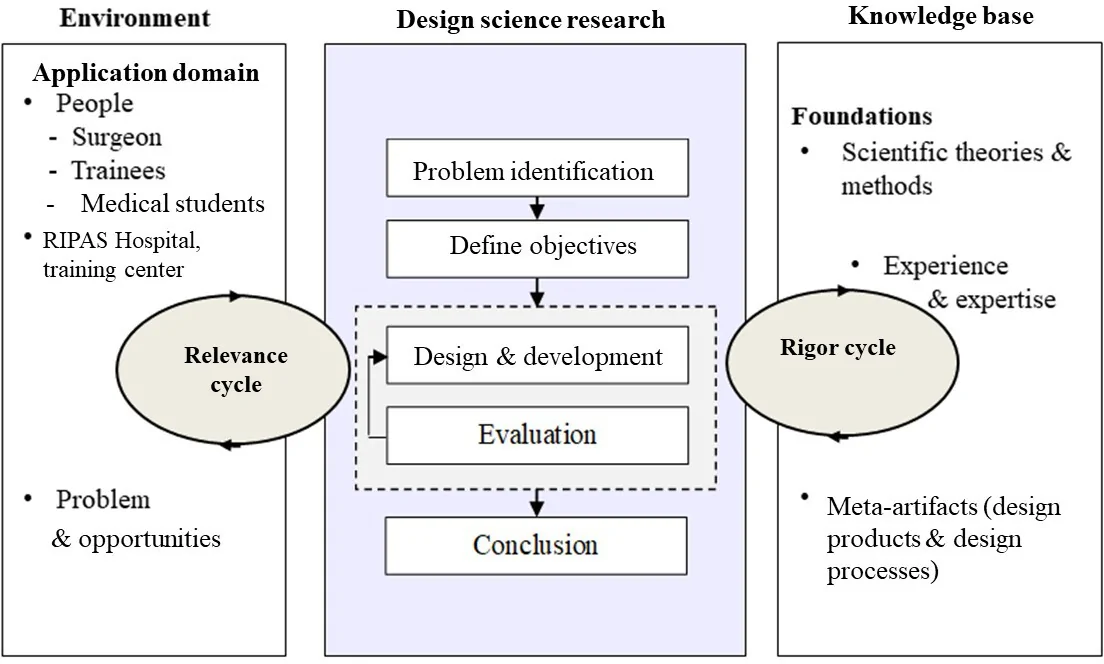
Adapted research framework [20]. RIPAS: Raja Isteri Pengiran Anak Saleha.

Design science research (DSR) is a problem-solving research paradigm that develops artifacts to address research challenges. It encompasses 2 primary activities: creating an artifact and evaluating its effectiveness in solving the identified problem [19]. In this study, the adapted design science research paradigm was applied [20], as shown in Figure 1. Throughout this research journey, multiple site visits and interviews with 6 medical experts were conducted at Raja Isteri Pengiran Anak Saleha (RIPAS) Hospital to examine current medical practices [21] and to discuss existing challenges. This paper documented the first iteration: curriculum analysis, mockup, and low-fidelity prototype development, and initial prototype assessment to gather expert feedback.

### Curriculum Analysis

A curriculum analysis [22] was applied to chapter 38, “Intussusception”, from Ashcraft’s Pediatric Surgery, Sixth Edition [23]. The analysis involved breaking down the chapter and evaluating the practical relevance of structural coherence. Consequently, 3 scenarios were identified. Table 1 presents the scenarios and concepts covered, along with their respective page numbers in the chapter.

**Table 1.** Mapping of the scenarios, subjects covered, and their corresponding page numbers.

Level	Name of scenarios	Concepts covered	Page number
Beginner	Early diagnosis and management of intussusception in a 9-month-old infant	Nonoperative management (pneumatic reduction)	531‐534
Intermediate	Delayed diagnosis of intussusception in a 2-year-old child	Nonoperative management (pneumatic reduction)	531‐534
Advanced	Complicated intussusception in a 3-year-old with peritonitis	Operative management (laparoscopic approach)	534‐537

### PRI: Low-Fidelity Prototype Development

Due to time constraints, the development of a low-fidelity prototype (illustrations) was based only on scenario one (Beginner) in Table 1. A pneumatic reduction procedure for children with intussusception was designed and illustrated [21] in Procreate (Savage Interactive) for the intended VR environment. The illustrations depicted the procedural workflow, expected user actions, and the positioning of relevant medical instruments within the simulation. As this prototype is the first iteration of the design, not all the interactions shown are implemented, but are planned. The system shall be developed subsequently as a VR application for the Meta Quest 3 headset, built in the Unity game engine. The low-fidelity prototype was used as a conceptual artifact to get early feedback from clinical experts.

**Figure 2.**
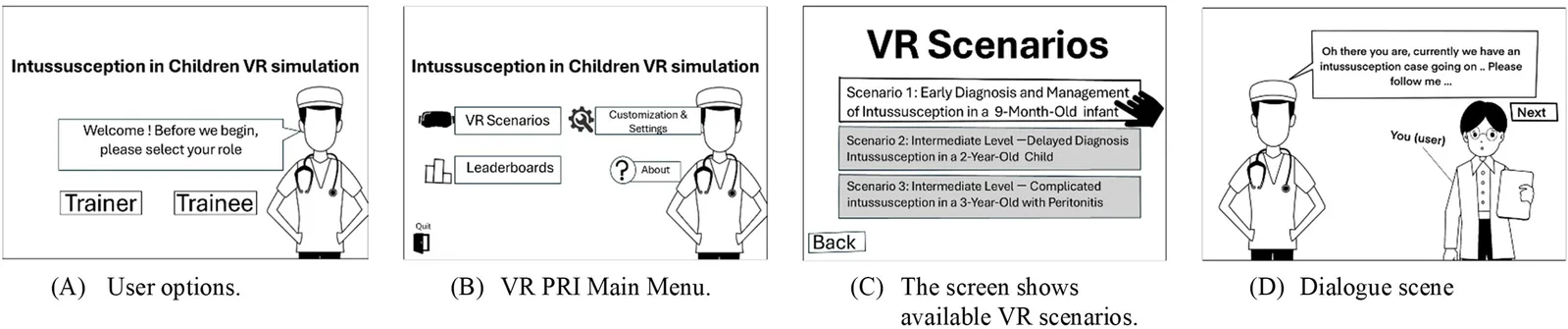
Interface of the system’s main menu. VR: virtual reality.

The main interface (Figure 2A) of the PRI prototype enables the user to select “Trainer” or “Trainee.” The “Trainer” option allows access to authoring tools (ATs) to customize and personalize the VR experience for trainees. This function requires further analysis and is outside the considerations of this study. The user is directed to the prototype’s simulated VR training interface (Figure 2B) by selecting the “Trainee” button. The trainee’s main menu consists of VR Scenarios, Leaderboards, Customization & Settings, and About. A virtual surgeon avatar is available in the main menu to assist via voice assistance.

In VR scenarios, trainees can select any simulation to practice, and Customization & Settings lets them customize the avatar and adjust audio or user interface (UI) settings. Leaderboards display the top 5 trainees ranked by accumulated points, with the top 3 receiving gold, silver, and bronze badges. The About option provides background information on the simulation, and Quit exits the program.

**Figure 3.**
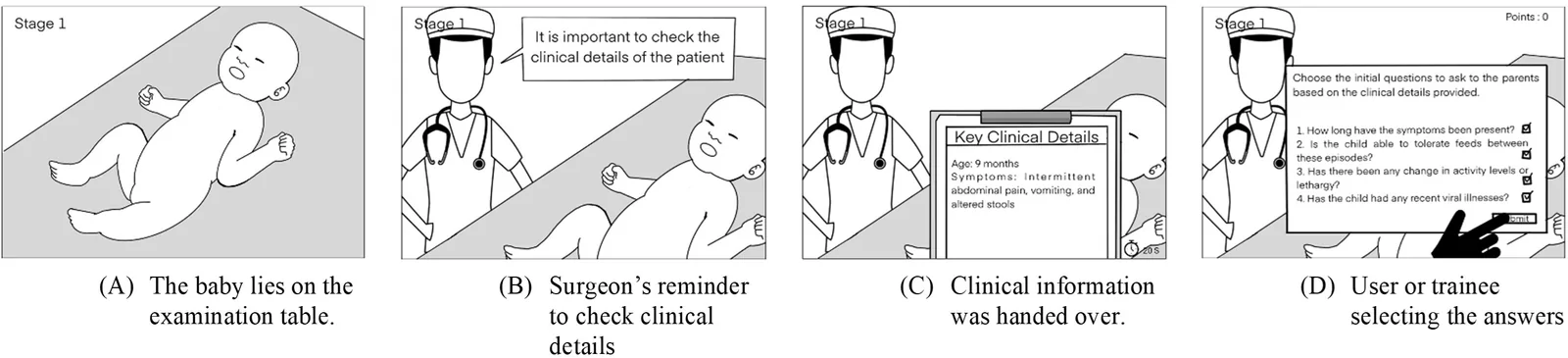
Scenario 1, Stage 1.

The user should choose between Scenario 1 and Scenario 3 in the VR Scenarios. Scenario 1 was implemented in the study (Figure 2C). When Scenario 1 was selected, a virtual surgeon welcomed the trainee with a narrative, intro, and objective (Figure 2D). The next button prompted the trainee to proceed to Stage 1 (Figure 3).

In Stage 1, the trainee views a 9-month-old female patient lying on the examination table (Figure 3A). Additional clinical details are provided via voiceover and subtitles (Figure 3B–C), which must be retained within a set time limit (eg, 20 s). After the information disappears, a question panel appears (Figure 3D). Trainees’ correct answers trigger the visual and audio feedback.

**Figure 4.**
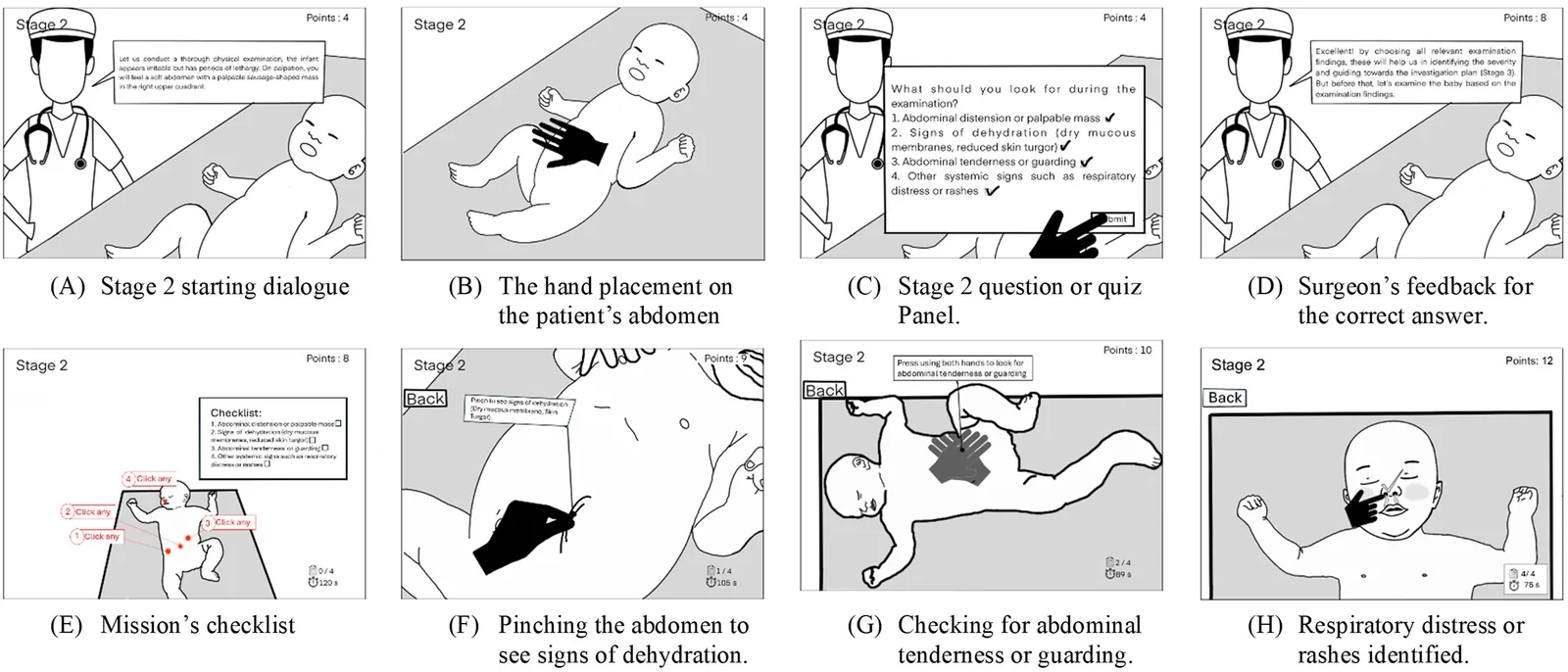
Scenario 1, Stage 2.

A total of 4 points are awarded for correct answers on the first attempt, after which the virtual surgeon provides feedback confirming accuracy and advances the trainee to Stage 2 (Figure 4).

At the start of the physical examination stage (Stage 2), the virtual surgeon initiates a dialogue, directing the trainee to palpate the child’s abdomen (Figure 4A). An arrow with an annotation then highlights the abdomen, prompting the trainee to begin the interaction. After initiation of the hand placement (Figure 4B), the trainee must wait a few seconds for completion, after which the Stage 2 question panel appears (Figure 4C). The virtual surgeon asks a multiple-response question, requiring all correct options to be selected before submission. Correct responses are awarded 4 points (Figure 4D). Afterward, the surgeon confirms accuracy and instructs the trainee to perform a physical examination based on the findings.

The checklist in Figure 4E appears floating in the environment, assigning the trainee a challenge or mission to examine the baby based on the reported findings, within a 120-second time limit. The trainee must identify 4 examination findings by interacting with the 4 numbered red circles. Each right answer earns a point. The trainee will advance to the subsequent stage of training even if the checklist was not completed within the assigned time.

**Figure 5.**
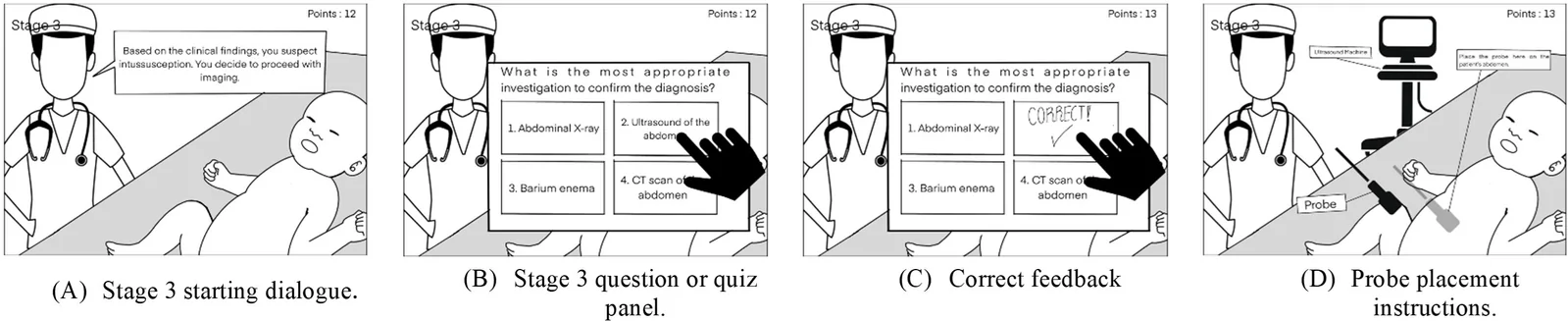
Scenario 1, Stage 3.

In Stage 2, the trainee interacts with the checklist by selecting the numbered red circles. Red Circle 2 assesses dehydration: pinching the baby’s stomach (Figure 4F) and releasing it will reveal reduced skin turgor, worth 1 point. Red Circle 3 directs the assessment of abdominal tenderness by applying pressure to the indicated area (Figure 4G); applying slight pressure causes the body to tense, earning 1 point. The Red Circle 4 prompts an examination of systemic signs, such as respiratory distress and rashes (Figure 4H). A point is awarded for the interaction, and the trainee is promoted to Stage 3 (Figure 5). The Return button returns the user to the previous camera view (Figure 4E).

**Figure 6.**
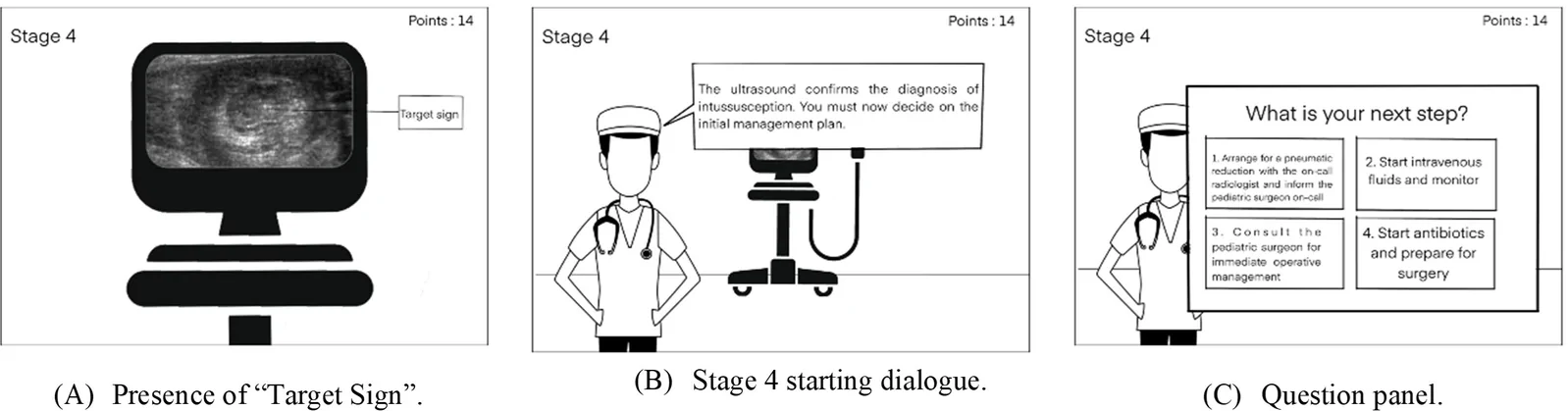
Scenario 1, Stage 4.

Through dialogue (Figure 5A), the virtual surgeon initiates Stage 3 (Investigation). A panel with questions for Stage 3 appears (Figure 5B). The trainee voices the answers or uses their finger to touch the screen. One selected answer per question (Figure 5C). Positive feedback is given when answers are correct, and corrective feedback is given for incorrect ones. After the trainee completes the quiz, the instructor informs the trainee that the investigation is an ultrasound and directs the trainee to place the probe on the patient’s abdomen (Figure 5D). This initiates the probe animation. At the end of the animation, the student clicks the ultrasound display to view the image. The initiation of Stage 4 (Management Plan; Figure 6) occurs upon completion of this task.

**Figure 7.**
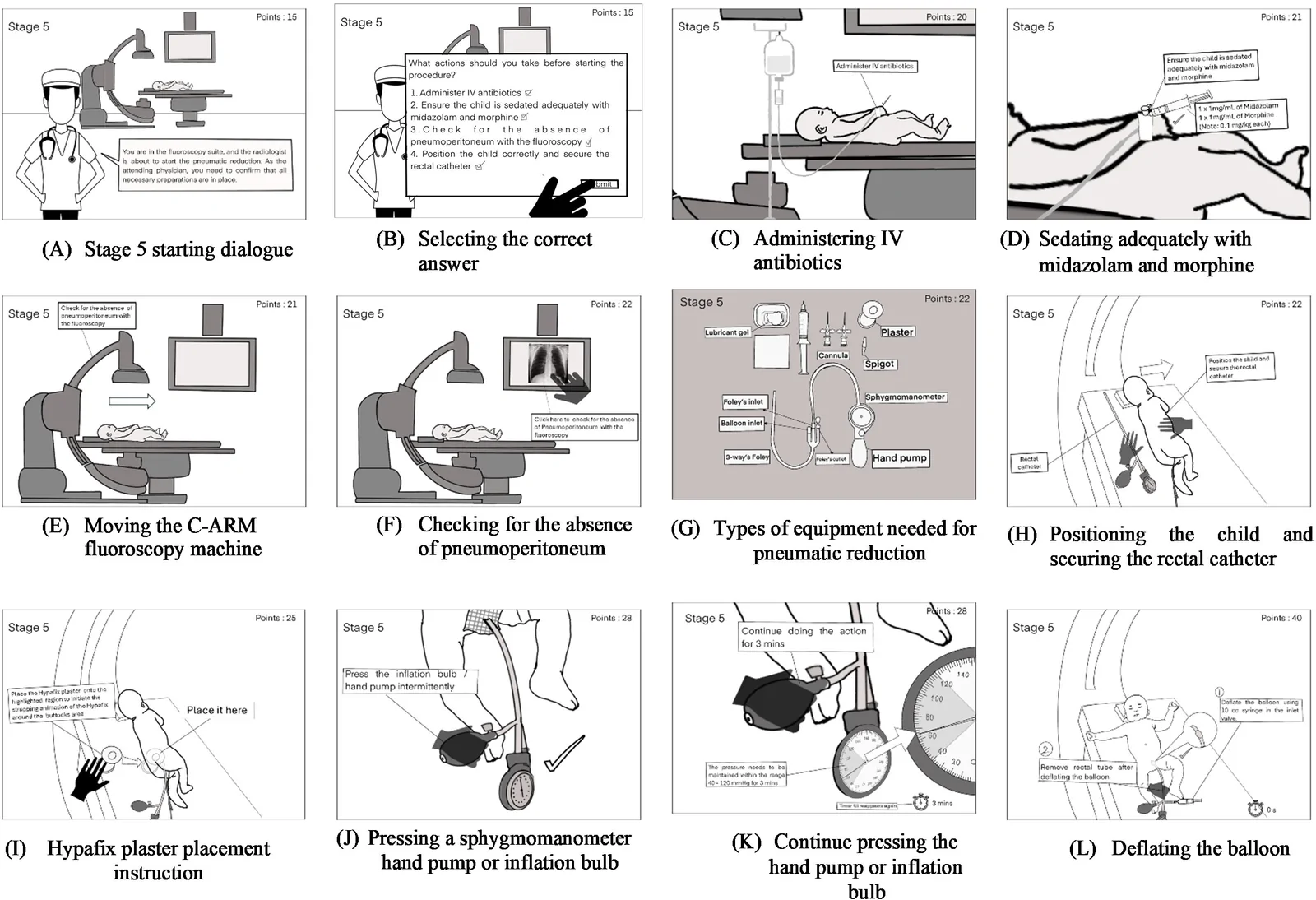
Scenario 1, Stage 5. IV: intravenous antibiotics.

Ultrasound images revealed the target sign (Figure 6A) indicative of intussusception. After that, the surgeon asks the trainee to select the treatment plan (Figure 6B), after which the Stage 4 question panel pops up (Figure 6C). The trainee chooses option 1: “Arrange for a pneumatic reduction with on-call radiologist and inform pediatric surgeon on-call” to earn 1 point. Once this level is completed, the trainee proceeds to Stage 5: pneumatic reduction simulation (Figure 7).

At the start of Stage 5, the virtual surgeon accompanies the trainee to the fluoroscopy suite and requests them to check the preparations (Figure 7A). Then, the question panel is displayed (Figure 7B). The trainee is asked a multiple-response question which requires them to select all options for 4 marks along with “CORRECT! Feedback coming afterward. As the surgeon suggests, everything is essential for the procedure to be performed safely. The trainee administers intravenous antibiotics (IV) via the cannula in the child’s wrist for 1 point (Figure 7C). The next step of the trainee is to position the child in the supine position. Midazolam 1 mg/mL and morphine 1 mg/mL, 0.1 mg/kg of each are to be given through the cannula for 1 point (Figure 7D). Subsequently, the candidate operates the fluoroscopy machine to evaluate for pneumoperitoneum (Figure 7E). The C-arm is adjusted by a radiologist, and the animated scan of the baby is proceeded with. The trainee then clicks on the X-ray result to confirm that there is no pneumoperitoneum (Figure 7F) to earn 1 point.

Prior to the simulation, the virtual surgeon informs the trainee that the equipment for the VR pneumatic reduction stage (Figure 7G) will be placed on the virtual table. To obtain 1 point, the trainee must position the child and secure the rectal catheter [21], which must be lubricated (Figure 7H). The syringe is filled with 20‐30 mL of air, and the trainee then injects the air into the balloon. Gently pulling the catheter to feel resistance scores one point. Finally, the buttocks are positioned and secured with Hypafix plaster in the color-coded area shown in (Figure 7I), which allows interaction in the animation.

**Figure 8.**
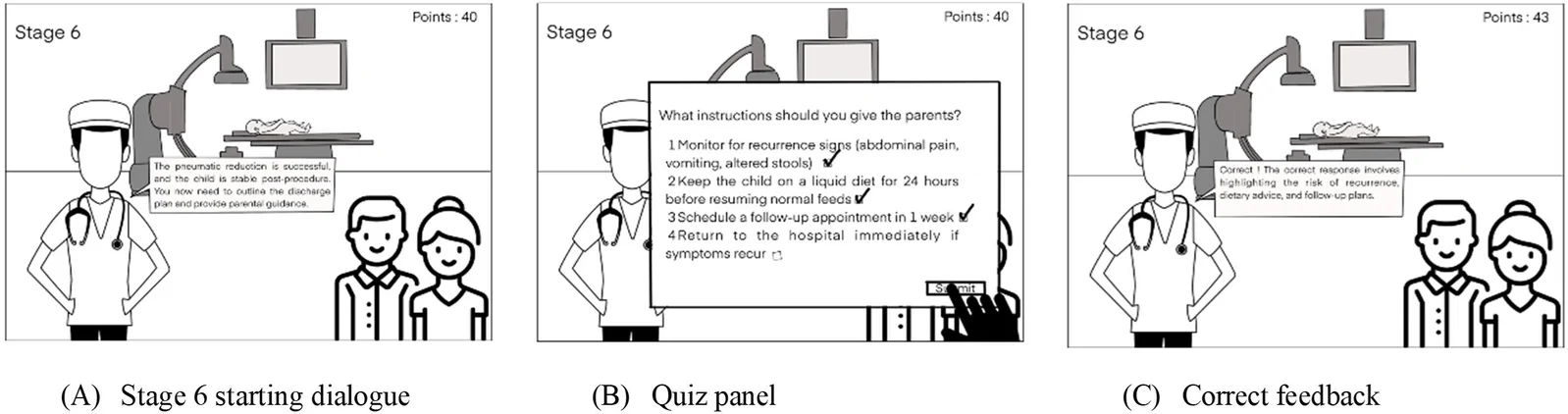
Scenario 1, Stage 6.

Once the Hypafix plaster is applied to the intended outline, the animation interaction starts. The student gets 1 point when the plaster looks well secured. The child is then placed supine on the fluoroscopy table, which gives you another point. The trainee next slowly and intermittently presses the sphygmomanometer hand pump to initiate pulsatile pneumatic reduction (Figure 7J) to get 1 point. The pressure should be maintained between 40 and 120 mm Hg for 3 minutes (Figure 7K). The timer UI must be monitored throughout. If the claw sign disappears, the trainee receives 10 points and may continue to maintain an estimated pressure of 40 mmHg for 30 seconds, which is another point confirming successful reduction. The Foley’s inlet is clamped after the balloon syringe is deflated and the spigot is opened (Figure 7L). This is scored as one, and the candidate goes to Stage 6 - Discharge Plan (Figure 8).

A reduction will be unsuccessful if the trainee fails to maintain pressure throughout the range and during further inflation, if the pressure exceeds 120 mmHg, perforation occurs, ending the scenario, and five points are awarded.

During Stage 6, the surgeon instructs the trainee to provide discharge instructions to the parents (Figure 8A). The Stage 6 question panel appears (Figure 8B), presenting a multiple-response question. The trainee selects all correct answers (1, 2, and 3) and submits; if correct, ‘CORRECT!’ feedback is displayed, and three points are awarded for a first-attempt correct response. The surgeon confirms the answers (Figure 8C), and a congratulatory message indicates that the scenario is complete. Points are recorded on the leaderboard.

### Method and Procedure

A focus group discussion was conducted with healthcare professionals involved in the management of pediatric intussusception as part of the evaluation phase. While high-fidelity VR development is expensive and time-consuming, a low-fidelity prototype was built and used in the focus group structured discussion. The prototype allowed participants to review the conceptual workflow and provide clinical insights to inform future VR system development.

The focus group discussion was conducted at a time and location chosen by the consultant pediatric surgeon (with 16 y of experience) from the Department of General Surgery at RIPAS Hospital. Other relevant medical staff were invited to participate in the focus group discussion (ranging from 5 to 10 y of expertise). Participants were purposively selected to represent the multidisciplinary team typically involved in the management of pediatric intussusception, including surgical, pediatric, nursing, and radiology perspectives.

Focus group questions are adapted from standard questionnaires, including the System Usability Scale (SUS), Usefulness, Satisfaction, and Ease of Use Questionnaire (USE), Computer System Usability Questionnaire (CSUQ), Questionnaire for User Interface Satisfaction (QUIS), Purdue Usability Testing Questionnaire (PUTQ), and Software Usability Measurement Inventory (SUMI) [24-29]. The questions were modified to remain relevant to the current research context.

After receiving adequate information from the researcher, participants provided informed consent to participate in the study. A clear explanation of their right to refuse to participate without penalty was part of the information shared with participants. The session lasted approximately 2 hours and 30 minutes. Table 2 presents the list of interview questions for the focus group and their corresponding categories.

**Table 2.** List of interview questions.

No. and categories	Questions
Category 1: pneumatic reduction: procedural steps, challenges, and current practices in Brunei	
Q1^a^	How many children have you managed for intussusception per wk/mo in Brunei? How frequent is it?
Q2	How many surgeons/medical staff are involved in the pneumatic reduction procedure?
Q3	Do you receive students for medical training at the hospital in Brunei?
Q4	How can we confirm that a student conducted this procedure correctly?
Q5	What aspects do you inspect when using tools such as rectal catheters?
Category 2: Formative evaluation of the PRI^b^ low-fidelity prototype and implementation of gamification guidelines	
Q6	Are the PRI procedures presented in the low-fidelity prototype easy to follow?
Q7	Are there any changes/improvements/suggestions that need to be made in the presented PRI low-fidelity prototype?
Q8	Is the interface of the low-fidelity uncomplicated/straightforward/easy to interact with?
Q9	Is the information presented in the low-fidelity prototype clear and understandable?
Q10	Are the low-fidelity graphics elements (icons, labels, buttons, and characters) clear, uncomplicated, and consistent across the prototype screens?
Q11	Is the terminology used in the low-fidelity prototype clear?
Q12	Are the buttons located at suitable locations?
Q13	Does the low-fidelity prototype have an attractive and pleasing design?
Q14	Do you like the system interface?
Q15	How important is it for trainees to use a prototype to familiarize themselves with the procedure before gaining clinical experience?
Q16	How do you determine whether the student/trainee has conducted the PRI-related task correctly or successfully?
Q17	Does using avatars for students/trainees interacting with the low-fidelity prototype enhance their motivation and engagement?
Q18	What do you think about using an avatar to represent a surgeon to guide and provide students/trainees with feedback? As shown in the low-fidelity prototype. Do you believe this can sustain students’ or trainees’ motivation?
Q19	Can providing students/trainees with tools to customize their avatar or setting, as shown in Figure 2B, improve their VR simulation experience, or could it distract their attention?
Q20	As a surgeon, do you think providing authoring tools to update the VR^c^ PRI experience based on students’ skills is necessary? As this allows the trainer to update the VR experience, do you think this is effective? Or should the students stick with the procedure exactly?
Q21	Do you believe rewarding *badges* for completing tasks could increase trainees’ motivation in PRI? What criteria should be considered for badge acquisition for this medical simulation?
Q22	What do you think about the challenges and quests implemented in the low-fidelity prototype? Is it engaging for medical trainees?
Q23	Do you think the three-scenario structure (Table 1), which will be implemented in the low-fidelity prototype, maintains engagement while ensuring effective learning?
Q24	Would the type of feedback (eg, auditory, visual, haptic) implemented in the low-fidelity prototype improve trainees’ motivation and learning outcomes within this simulation?
Q25	Do you think implementing *leaderboards* in the low-fidelity prototype motivates medical trainees to engage more actively in the simulation?
Q26	Would competition through leaderboards be beneficial, or could it create unnecessary pressure and reduce motivation?
Q27	Do you think the narratives/storytelling implemented in the low-fidelity prototype motivate students in the medical simulation?
Q28	Do you think the scoring system implemented in the low-fidelity prototype is effective?
Q29	Can applying quizzes motivate students to engage in this simulation?
Q30	Would incorporating a multiplayer or collaborative element into the ’Intussusception in Children’ VR simulation improve engagement and motivation for trainees?
Q31	How do you see VR’s role in enhancing motivation and engagement in procedural training? Could it place students under pressure?
Q32	Do you think that implementing time limits in this simulation could enhance trainees’ motivation and focus?
Q33	Which style do you prefer for the simulation: realistic or cartoonish?
Q34	Are there any additional aspects of the low-fidelity prototype that you would suggest improving?
Q35	Do you agree that implementing the guidelines in the “Intussusception VR simulation” prototype can increase trainee motivation and engagement?

a Q: question.

b PRI: pneumatic reduction of intussusception.

c VR: virtual reality.

### Participants

The focus group comprised 6 participants, including 1 pediatric surgeon, 1 pediatrician, 2 nurses, and 2 radiographers. Table 3 presents the categories of participants included in the focus group.

**Table 3.** List of participants.

Role	Participant ID
Surgeon	P1
Pediatrician	P2
Nurse	P3-P4
Radiographer	P5-P6

### Ethical Considerations

Ethical approval was obtained from Universiti Teknologi Brunei Institutional Review Board and Research Ethics Committee (approval no. UTB-URC/IRB/SCI/2026/005). The research project conformed to Universiti Teknologi Brunei’s ethical requirements of study. A low-fidelity VR prototype was evaluated in a focus group discussion with 6 health care professionals. Informed consent was obtained from all participants in writing. Participation was voluntary, and participants could withdraw at any time without consequence. All data before analysis were anonymized.

## Results

### Overview

In this section, we present the findings from a focus group discussion involving a surgeon and medical professionals regarding the low-fidelity prototype. The focus will be on procedural and design aspects, as well as suggestions for creating a high-fidelity VR experience. The results are organized by the categories listed in the interview protocol (Table 2).

### Pneumatic Reduction: Procedural Steps, Challenges, and Current Practices in Brunei

In response to a question on how many pediatric intussusception cases are managed per year in Brunei (Question 1), the participant (P1) reported managing between 9 and 14 cases per year, most of which occur during the mid-year months (May to August). P1 also indicated which staff are useful for doing or assisting the pneumatic reduction (Question 2). This is usually a surgeon, pediatrician, nurse, and radiographer. Understanding team dynamics enables the simulation to replicate real-world clinical practice [21]. In response to the question about whether participants P1 and P2 supervise medical students at the hospital (Question 3), they said that “Yes, from time to time, we have medical students from the Universiti Brunei Health Institute and postgraduate students looking for exposure to pediatric surgical cases.”

In addition, to ensure the trainee can perform successfully (Question 4), participants (P2, P4, and P6) asserted that the trainee must be well-versed with each step and scenarios before touching the patient as a safety precaution. For example, trainees must understand that the catheter must be inserted 10 cm into the rectum before inflating the balloon, as failure to do so can cause complications (Question 5).

### Formative Evaluation of the PRI Low-Fidelity Prototype and Implementation of Gamification Guidelines

In reviewing the low-fidelity prototype, participants (P1 and P2) provided insights on implementing the VR simulation and the gamification guidelines. Ensuring procedural accuracy in the prototype is critical to its educational effectiveness. As shown in Table 3, participant (P1) indicated that the procedural steps were easy to follow (Question 6).

In response to Question 7, several adjustments were suggested by the participants (P1 and P3) to improve realism and ensure alignment with clinical practices in Brunei [21]. For example, the sequence of steps in the physical examination was refined to provide a more logical progression. Certain figures, such as Figure 4E, were recommended for removal, as the physical examination should commence earlier (Figure 4B). Furthermore, the surgeon (P1) emphasized that the depiction of examination findings in Figure 4E should be included to provide a more comprehensive summary, incorporating relevant clinical indicators such as absence of dehydration, right-sided intraabdominal mass, mildly distended but soft abdomen without guarding, and absence of respiratory distress or rashes.

Additional refinements were suggested by a participant (P1) regarding the assessment of skin turgor in the abdominal region (Figure 4F), which was recommended for exclusion because it is controversial and should not have been performed there. Instead, this assessment should be conducted on the hands for greater accuracy. Similarly, modifications were proposed to depict guarding assessments (Figure 4G), in which a single-hand technique was advised rather than using both hands. For Figure 7C, participants (P1, P2, P3, and P4) noted that in actual clinical practice, intravenous fluids would have already been prepared before the procedure. Therefore, they highlighted that this step should not be simulated as part of the procedure.

Furthermore, the participants (P1 and P2) highlighted that the image representing the absence of pneumoperitoneum (Figure 7F) was inaccurate; a corrected version was later provided by the participants after the interview.

Equipment considerations specific to Brunei’s medical context for pneumatic reduction were highlighted, including the standard use of 2-way Foley catheters rather than three-way Foley catheters, thereby eliminating the need for a spigot and cannula (Figure 7G). The participants (P1, P2, P3, and P4) also noted that the low-fidelity interface was straightforward and easy to interact with (Question 8). They confirmed a clear understanding of the information presented in the prototype (Question 9) and the terminology used (Question 11).

In addition, they acknowledged the consistency of graphic elements across the screen (Question 10), the appropriate placement of buttons (Question 12), and the attractive, user-friendly design (Question 13). Overall, they expressed a positive impression of the system interface (Question 14). However, participants (P5 and P6) raised concerns regarding the placement and functionality of the Back button (Figure 4F–H). They noted that, within a VR environment, the presence of a Back button at this stage may disrupt immersion. Therefore, they recommended removing the Back button to encourage trainees to physically navigate the virtual environment, thereby enhancing immersion, rather than relying on a clickable navigation option in the 3D space.

When asked about the importance of using a prototype to familiarize trainees with the procedure before gaining clinical experience, the surgeon (P1) emphasized its value, stating, “It is vital for the trainee to have such a prototype to familiarize himself/herself with the procedure and understand the concept, which will expedite the attainment of clinical experience” (Question 15).

The participants, P5 and P6, also noted that the trainees need to be familiar with the VR system’s controls. As a result, they proposed that the researcher provide a briefing before the session to avoid misunderstandings and conduct the training.

According to the surgeon, P1’s statement in Question 16 defined simulation success for pneumatic reduction. The fluoroscopic view of regression of the intussusception, the disappearance of the claw sign, and free air entry into the small bowel were the identifiers of the success as stated by the surgeon. The criteria prepare trainees to be competent in assessing procedural outcomes in the simulation.

Considering avatars in the children’s intussusception VR simulation (Question 17), (P1, P2, P3, and P5) shared that they will enhance motivation and engagement because this “relatable” digital representation (Figure 2D) creates connectedness, which enhances the sense of being (spatial presence) and social presence. One participant noted that the surgeon’s avatar could serve as an agent and mentor (Question 18), supporting performance and providing feedback in a less antagonistic manner, thereby enhancing trainees’ comfort with making mistakes. Also, it was indicated that allowing trainees to customize their avatars might add fun and further increase engagement (Question 19). The participants (P4 and P6) suggested that the prototype’s main interactions should be prioritized first in this iteration. It is okay to accept customizable avatar options, but a default avatar will also suffice at this stage. They said the customization feature should be grayed out so trainees know it will be available in the next phase.

The majority of respondents believe that ATs in a medical training environment could enhance trainees’ learning experiences (Question 20). For instance, as illustrated in Figure 2A, which was approved without objection, the “Trainer” button enabled trainers to customize content for students as needed. According to them, awarding badges for task completion can heighten motivation and engagement (Question 21). On the contrary, they claimed that the stage low-fidelity prototype did not outline badge criteria. The badges should not only be for the top 3 winners but also include badges for completing the scenario, answering questions correctly, or performing procedures accurately in time.

In addition, the participants (P1 and P2) expressed their acceptance of the challenges and quests set in the simulation to engage the medical trainees (Question 22). According to the information obtained, the VR prototype includes different scenarios (Question 23), each designed as a level with its own challenges and tasks, where trainees must complete the tasks to advance to the next level, thereby introducing a sense of progress & achievement. They also stated that the time limit (Question 32) could be causing challenges, as it imposes pressure, as shown in Figures 4E , and 7K, denoted by the stopwatch icon at the bottom-right of the screen.

On the contrary, in response to Question 24, participants P1, P3, and P4 stated that the nature of feedback most likely to improve trainees’ learning outcomes depends on the effectiveness of the VR simulation. They recommended combining different types of feedback, such as visual and auditory (for example, sound effects for correct and incorrect answers; Figure 6C), a surgeon avatar providing guidance with mixed feedback (Figure 3B), and haptic feedback for added realism (for example, during physical examination, staying with the abdominal mass to improve realism (Figure 4B). Consequently, rather than prioritizing any one type of feedback, they strongly encouraged combining multiple sources of feedback to improve the simulation.

Regarding the leaderboard, the surgeon (P1) stated that it will be beneficial for trainees who like competition (Question 25) but may also make other trainees anxious and less intrinsically motivated (Question 26). He warned that trainees would be more concerned about rankings than the content of learning itself. Participants were advised to use a tracker to monitor their progress rather than relying on others’ scores (P2 and P3). The adoption of a leaderboard should be carefully considered to prevent adverse impact.

From the participants’ perspective (P1 and P2), it was acceptable to use storytelling and narratives (Question 27) in the gamified medical simulation, as they could effectively engage trainees with the learning materials. As illustrated in Figure 4A, the simulation describes a child patient with symptoms. The use of storytelling through narratives was found to be effective in building empathy, making learning less abstract and more memorable, meaningful, and realistic.

Participants favored including the scoring system (Question 28). Trainees earn points for answering questions or taking actions correctly, and lose points for mistakes. This will provide trainees with opportunities to gain experience and learn from mistakes. The addition of quizzes (Question 29) could serve as a motivator as it provides instant feedback, but the surgeon (P1) stated that they should be related to the story to enhance immersion. As illustrated in Figure 4C, quizzes that allow for interactive dialogues will help trainees engage with the content and contextualize procedural steps.

P1, P2, and P6 stated that the use of these guidelines could bring a positive impact on trainee motivation (Question 35). However, the multiplayer and social support elements did not work well (Question 30) because collaborative decision-making is usually not part of pneumatic reduction.

Participants (P1, P3, P4, and P6) were asked to rate VR’s potential to increase motivation and engagement during procedural training (Question 31). In their opinion, the technology has the potential since learners can make mistakes without real-world consequences. From their perspective, VR-based learning in pneumatic reduction for intussusception procedures is more motivating than learning from a textbook.

The visual and interactive design of the low-fidelity prototype is assessed for its feasibility for trainee engagement and comprehension. Despite the surgeon (P1) requesting flexibility in graphic styles for the high-fidelity prototype (Question 33), a series of recommendations were made to improve the experience and narrative (Question 34). One important recommendation was to redesign the simulation to configure scenarios as levels rather than separate cases (Figure 2C), which aligns with gamification principles and supports progressive learning. The dialogues should also be revised for clarity and engagement. For instance, the first dialogue (Figure 2D) was modified to allow for better immersion: “Welcome to the management of intussusception VR simulation. Are you ready?” Moreover, the original dialogue of the scenario was edited to ensure trainee engagement: “Oh, there you are. We currently have a 9-month-old baby to review. Please follow me.”

Participants (P1 and P2) suggested further refinements to the simulation’s interactive assessments. In the quiz part (Figure 3D), the second question should be added as “How long does each episode of a pair last, and what is the interval between episodes?” Likewise, in Figure 4C, they proposed substituting “reduced skin turgor” with “presence of tears”. In Figure 8B, the surgeon (P1) suggested refinements to the postoperative management instructions to better reflect clinical practice as follows:

1. Monitor for recurrent abdominal pain.
2. Remain in the ward for 48 hours before discharge.
3. Repeat ultrasound if recurrence is suspected.
4. Allow feeds after 4 hours.

These alterations ensure that trainees also gain a clearer understanding of posttreatment and reinforce the knowledge and skills used to implement the pneumatic reduction.

## Discussion

### Principal Findings

The research illustrates how the gamification guidelines can be translated into a VR simulation that structures procedural learning in pneumatic reduction training.

The results show that certain game elements, such as avatars and storylines, can be incorporated into a VR simulation to reduce intimidation and enhance motivation and engagement in the learning environment. Also, the results revealed that user-friendly ATs could enhance learning experiences by helping the trainer tailor content to the trainee’s needs. In addition, clinical specialists confirmed that the prototype accurately represented the key steps and could engage learners during simulation-based education. Furthermore, they agreed that the prototype could also serve as a preclinical familiarization tool, allowing a trainee to learn about a concept before entering the clinical setting and applying it to a patient. These findings are similar to those of previous studies, which have indicated the effects of gamification on learners’ motivation and engagement. Previous research has concluded that VR simulation can be useful for procedural understanding in medical discipline training. For instance, Yang et al [9] found that compared with passive VR and traditional videos, gamified VR leads to better preparedness for MRI among adolescents.

On the other hand, most participants agreed that instance feedback should not rely solely on a single source and strongly recommended combining multiple sources, such as visual and auditory feedback. Participants recommended treating the VR scenarios as levels to support progressive learning and provide a sense of achievement. Moreover, all participants favored adding the scoring systems, as it allows trainees to gain experience and learn from mistakes. Some participants agreed that using quizzes could be a motivator, as it provides immediate feedback.

The result revealed that incorporating the time limit is not recommended, as it could pose pressure on the trainer during the learning experience. In addition, the results showed that the low-fidelity system’s interface design is clear and straightforward. The results indicated that although the leaderboard can be motivating for learners, it’s not recommended for the PRI simulation because it introduces pressure. In addition, most participants agreed to award badges to the trainee for task completion, and the badge-acquisition criteria need to be outlined. Finally, participants do not recommend incorporating multiplayer into the prototype, as collaborative decision-making is not usually part of pneumatic reduction.

### Limitations

Although low-fidelity prototypes offer valuable insights, they have some limitations. Initially, the prototype is the first version developed in close cooperation with surgeons and medical experts to generate clinical requirements and obtain expert review of the conceptual design. At this point, a high-fidelity prototype would be costly and labor intensive to produce. Furthermore, the evaluation of the low-fidelity prototype was conducted using a non-interactive VR simulation system representing the design concept. The prototype measurement did not involve learning outcomes assessment, skill performance assessment, or clinical assessment. The next phase of work will produce a high-fidelity VR prototype and test it with a larger, more diverse group of participants, including Bruneian medical students. This would facilitate the assessment of the effectiveness of gamified learning approaches and improve the generalizability of the results obtained. Moreover, the prototype can be expanded to encompass two additional clinical scenarios identified through curriculum analysis, thereby increasing its capacity to train pediatric surgeons and medical trainees.

### Conclusions

This study explored how gamification guidelines are applied in the design of a VR simulation for pneumatic reduction of intussusception in children. The preliminary findings from clinical experts’ development and formative evaluation of a low-fidelity prototype indicate how selected game-based design principles may be implemented in a procedural training environment while remaining clinically aligned. The findings suggest that gamified VR simulations can effectively aid preclinical familiarization in pediatric surgery. The presented evidence can assist research in developing more engaging simulation-based learning and provides a strong base for developing and evaluating gamified VR training systems for pneumatic reduction in a subsequent stage. Future work will include a high-fidelity prototype, along with assessing the prototype with a large number of participants.

## Abbreviations

AR: augmented reality
AT: authoring tool
CSUQ: Computer System Usability Questionnaire
DSR: design science research
IV: intravenous antibiotics
MRI: magnetic resonance imaging
PRI: pneumatic reduction of intussusception
PUTQ: Purdue Usability Testing Questionnaire
QUIS: Questionnaire for User Interface Satisfaction
RIPAS: Raja Isteri Pengiran Anak Saleha
SUMI: Software Usability Measurement Inventory
SUS: System Usability Scale
UI: user interface
USE: Satisfaction, and Ease of Use Questionnaire
VR: virtual reality
